# Human Auditory Cortical Activation during Self-Vocalization

**DOI:** 10.1371/journal.pone.0014744

**Published:** 2011-03-03

**Authors:** Jeremy D. W. Greenlee, Adam W. Jackson, Fangxiang Chen, Charles R. Larson, Hiroyuki Oya, Hiroto Kawasaki, Haiming Chen, Matthew A. Howard

**Affiliations:** 1 Department of Neurosurgery, University of Iowa, Iowa City, Iowa, United States of America; 2 Department of Communication Sciences and Disorders, Northwestern University, Evanston, Illinois, United States of America; The University of Western Ontario, Canada

## Abstract

During speaking, auditory feedback is used to adjust vocalizations. The brain systems mediating this integrative ability have been investigated using a wide range of experimental strategies. In this report we examined how vocalization alters speech-sound processing within auditory cortex by directly recording evoked responses to vocalizations and playback stimuli using intracranial electrodes implanted in neurosurgery patients. Several new findings resulted from these high-resolution invasive recordings in human subjects. Suppressive effects of vocalization were found to occur only within circumscribed areas of auditory cortex. In addition, at a smaller number of sites, the opposite pattern was seen; cortical responses were enhanced during vocalization. This increase in activity was reflected in high gamma power changes, but was not evident in the averaged evoked potential waveforms. These new findings support forward models for vocal control in which efference copies of premotor cortex activity modulate sub-regions of auditory cortex.

## Introduction

During normal human speech, speakers modulate their vocalizations to adjust to environmental conditions. For example, during phonation, altering the pitch of real-time auditory feedback a speaker receives results in alterations in the produced voice fundamental frequency [1]. In order to accomplish this task, the speaker must be able to distinguish between self-generated vocalizations and externally generated sounds. The ability to discriminate between these two categories of stimuli is hypothesized to be dependent upon a feedback system of functionally connected brain regions involved in both the production and perception of speech [2]. A variety of experimental strategies have been used to explore the neural basis of this system and test theoretical models of vocal motor-sensory integration [3], [4], [5], [6], [7], [8], [9], [10]. One such approach examines how the act of vocalization influences brain processing of self-generated sounds. In the vocalization-playback experiment auditory brain responses are measured during vocalization, and then compared with responses obtained when the subject listens to a recording of these same vocalizations [11], [12].

To date, investigators have exclusively used non-invasive methods to measure brain activity in human subjects during vocalization-playback experiments using the subjects' own voice. Scalp electroencephalographic (EEG) and magnetoencephalographic (MEG) recordings have shown a reduction in the amplitude of auditory evoked responses when subjects vocalize compared to when they quietly listen to a recording of these same vocalizations [3], [4], [5], [6], [7], [8], [9], [10]. Functional neuroimaging studies performed using fMRI and PET methods also report a reduction in the activation of temporal lobe auditory cortex during vocalization compared to vocal playback [13], [14], [15], [16], [17], [18]. These findings of an inhibitory effect within auditory cortex during human vocalization are consistent with the results of earlier experimental animal studies [19], [20]. Such an inhibitory effect is predicted by forward models of sensory-motor integration, whereby brain responses are attenuated when the auditory stimulus the subject hears matches the intended vocalization generated by the motor system [21].

The current experiments were carried out in order to directly measure the effects of vocalization on speech-sound processing by taking advantage of the high spatial resolution of implanted intracranial electrodes in neurosurgery patients undergoing epilepsy surgery. By recording evoked brain activity from electrode arrays placed on the lateral superior temporal gyrus (STG) it is feasible to study electrophysiological activity from auditory cortex with a combined spatial-temporal resolution that cannot be achieved using non-invasive methods. We used this recording approach during vocalization-playback experiments to test the hypothesis that vocalization-associated changes in speech sound processing occur mainly within localized areas of human auditory cortex and the overall nature of these changes would be attenuation. This hypothesis, which is an element of some forward models [21], is based on the assumption that vocal motor control regions in human frontal lobe route an efference copy of motor commands to temporal lobe auditory cortex in a field-specific manner [22], [23], [24].

## Materials and Methods

### Subject Selection and Electrode Implantation

The subjects (N = 10) in this report were patients (5 male, 5 female) undergoing surgical treatment of medically intractable epilepsy who volunteered to participate in this research protocol. Their ages ranged from 20 to 62 years (mean 35.6 yrs). Written informed consent was obtained from each subject and all research protocols were approved by the University of Iowa Human Subjects Review Board. Subjects did not incur any additional medical risks by participating in these studies.

Each subject completed an extensive pre-surgical assessment including detailed neurological examination, brain imaging (MRI, PET, and SPECT), and neuropsychological evaluation. These tests confirmed normal speech and language functions in all subjects. No anatomic lesions were observed in the frontal lobe or temporal lobe auditory cortex in any subject. Standard audiometric testing was conducted and all patients were found to have normal hearing. All but one subject underwent preoperative sodium amobarbital (WADA) testing [25] to determine hemispheric language dominance. The left hemisphere was dominant for language in eight subjects and bilateral language representation was noted in two subjects. The subject that did not undergo WADA testing was strongly right-handed and for the purposes of this report was presumed to have left cerebral dominance for language. Experiments were conducted in a specially designed and electromagnetically-shielded private patient suite in the University of Iowa General Clinical Research Center.

**Figure 1:**
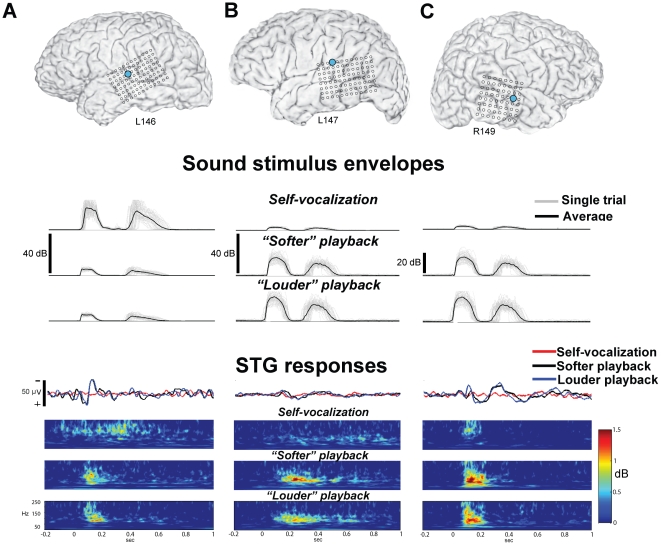
Vocalization-associated changes in auditory responses are not significantly altered by changes in PB intensity. Different intensities of the PB stimuli were tested in the three subjects shown. Each vertical column displays a recording site location (top, filled blue circle), sound stimulus envelope tracings (middle) and the evoked responses recorded from the selected recording site (bottom) for each subject. (A) Subject 146 perceived the PB stimuli to be both “softer” and “louder” than the SV stimuli despite the fact that the sound stimulus envelope was smaller at both PB intensities than those measured during SV. The AEP waveform is nearly identical for the ‘softer’ and ‘louder’ PB stimuli, and is completely attenuated during SV. The high-gamma (HGB) response shows a ‘sustained’ pattern during SV, and an ‘on’ pattern during both PB conditions, with the early HGB increase seen during PB to be attenuated during SV. Subjects 147 (B) and 149 (C) both perceived the PB stimuli to be both “softer” and “louder” than the SV stimuli yet for these subjects the measured sound stimuli envelopes were greater for both PB intensities compared to that measured during SV. Like subject 146, both subjects demonstrate attenuation of the AEP and HGB power responses during SV compared to both PB intensities, and little difference is seen in AEP and HGB power responses between the PB intensities.

As part of a standard multi-disciplinary epilepsy surgery evaluation and treatment protocol, each subject was deemed to be an appropriate candidate for surgical placement of intracranial multi-contact recording arrays for the purpose of recording and anatomically localizing seizure events. During an implantation operation, custom manufactured high-density electrode arrays (see below) were placed on the pial surface of the exposed brain regions. The electrodes remained in place during a 14-day hospital stay during which time the patients underwent continuous video-EEG monitoring. This high-resolution EEG monitoring confirmed that the peri-Sylvian cortical areas pertinent to this study (e.g. posterior inferior frontal gyrus, lateral peri-Rolandic cortex, STG) did not show abnormal inter-ictal activity. At the completion of the monitoring period, the electrodes were removed and the seizure focus was resected. Resections in all 10 cases were restricted to the anterior temporal pole and mesial temporal lobe structures. The resections did not involve the STG. The surface recording arrays consisted of platinum-iridium disc electrodes embedded within a silicon sheet with 5 mm center-to-center spacing and 3 mm contact diameter (Ad-Tech, Racine, WI). In eight subjects the high-density recording grid consisted of 96 contacts, while one had a 64-contact high-density grid. One subject received a 32-contact low-density grid (1 cm center-to-center contact spacing). Separate electrodes were implanted in the subgaleal space over the vertex to serve as reference contacts. The exact position of each recording electrode was localized using a combination of high-resolution digital photographs taken intra-operatively during electrode placement and removal, as well as thin-cut (1 mm) pre- and post-implantation MR and CT scans. Pre- and post- implantation MRIs were co-registered using a 3-D rigid-fusion algorithm implemented in Analyze software (Biomedical Imaging Resource, Mayo Clinic) [26]. Coordinates for each electrode obtained from post-implantation MRI volumes were transferred to pre-implantation MRI volumes. The location of every contact relative to visible surrounding brain structures was compared in both pre- and post-implantation MRI volumes. Such comparisons are useful since implantation of surface electrodes displaces the cerebral hemisphere medially with superficial brain tissue being distorted more than deeper structures. The resultant electrode locations were then mapped to a surface rendering of the lateral cerebral convexity (e.g. Figs. 1A, B, C). We estimate that the overall error in electrode localization using these techniques does not exceed two mm [27].

### Auditory Stimulus presentation

Acoustic stimuli were presented during two separate sessions; a self-vocalization (SV) session and a passive listening (playback, PB) session. For both sessions, the subject was resting comfortably in their hospital bed or a recliner. During the SV session, each subject was instructed to speak the same utterance (e.g. “birthday”) in a consistent manner using a normal, conversational speech intensity and rate, with an approximate two second interval between utterances. The entire vocalization session, consisting of approximately 50 utterances, was captured and recorded using a microphone (Shure beta 87, Niles, IL) held by the subject approximately one inch from their mouth using the hand ipsilateral to the brain hemisphere from which recordings were obtained. In the PB condition, the recorded utterance was played back via a pair of headphones (Etymotic ER4, Elk Grove Village, IL) placed in custom-fit, vented insert ear molds. The subjects heard their own vocal production that was amplified (10 dB, Mark of the Unicorn, Cambridge, MA), passed unaltered through a harmonizer (Eventide Eclipse, Little Ferry, NJ) and routed back to the headphones. The harmonizer was controlled with MIDI software (Max/MSP v4.5, Cycling '74, San Francisco, CA) by a standard laboratory computer. The contribution of bone conduction during the SV block cannot be measured or manipulated; therefore we used a strategy employed by previous investigators to determine whether the sound intensity of stimuli could account for any observed differences in evoked responses [28], [29], [30]. In the first 3 subject's experiments, we examined the effects of differing sound intensities during PB. Each of these 3 subjects were asked to complete 2 PB blocks; one block utilized a sound intensity adjusted by the subject to a level such that they described the PB sound intensity as “less than” the intensity of the sound they produced during the SV block. The second PB block utilized a sound intensity level that each subject described as being “greater than” the sound intensity of their own utterances during the SV block. Data showing the sound intensity levels selected by these three subjects for the ‘softer’ and ‘louder’ PB conditions are displayed in figure 1. Analysis of responses obtained using these different intensity settings for the PB stimuli showed no significant changes in the overall pattern of responses. Since vocalization-associated auditory cortical effects were not significantly altered by these changes in the sound intensity of the PB stimuli (Fig. 1), no further manipulations of PB sound intensities were performed in subsequent subjects. These later subjects adjusted PB intensity such that they subjectively perceived the intensity of the PB stimuli to be equal to the sound intensity they experienced during the SV block.

### Electrophysiology recording

Details of the electrode implantation method and data acquisition techniques used have been described previously [31], [32]. Briefly, auditory average evoked potentials (AEPs) were continuously recorded via electrode arrays (see above) implanted on the pial surface overlying the peri-Sylvian region of the temporal and inferior parietal lobes. The exact position of the recording grid differed somewhat between subjects as grid placement was determined based on clinical considerations for each subject. In all subjects, the coverage provided by the array included significant portions of the STG, including a previously described posterior lateral superior temporal auditory area (PLST) [31]. Arrays were located in the left cerebral hemisphere in 6 subjects, and in the right hemisphere for 4 subjects. For purposes of this study, electrode contacts outside of the region of interest (temporal lobe auditory cortex) were not included in the analysis. Research recordings were initiated several days post-implantation, after subjects had fully recovered from implantation surgery. AEPs were acquired using a TDT system (Tucker Davis Technologies System3, Alachua, FL) under both SV and PB conditions. Signals were filtered (1.6–1000 Hz) and digitized on-line (2034.5 Hz). Digitized data were stored for later offline analysis using MATLAB software (Mathworks, Natick, MA). Local field potentials were examined using conventional averaging methods as well as with techniques for measuring frequency band specific power changes.

### Data analysis

**Figure 2:**
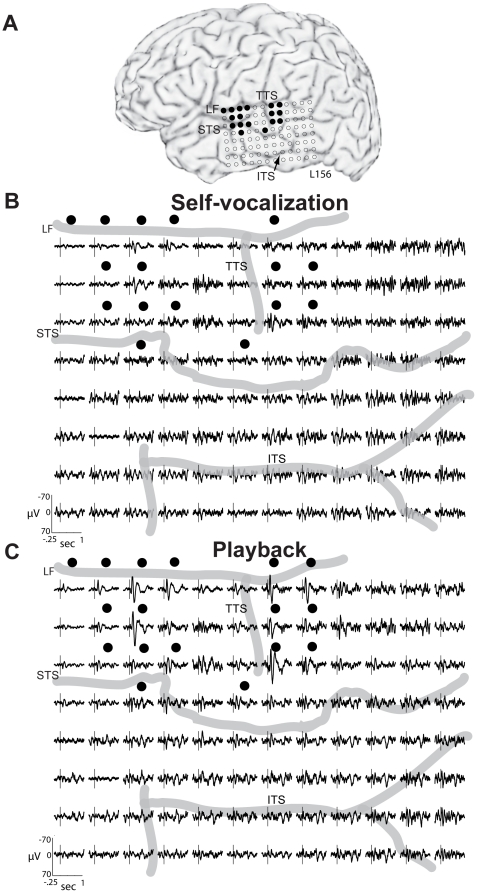
Averaged evoked potentials recorded from subject 156 during self-vocalization and playback. (A) MRI surface rendering of the subject's left hemisphere demonstrating the location of the 96 contact recording array. Filled black circles denote contacts where the AEPs recorded during SV were attenuated (p<.01, 0–500 msec post-stimulus) compared to the AEPs recorded during the PB condition. (B) AEPs recorded from the lateral surface of the cerebral hemisphere during SV. The timing of vocalization onset is represented in each waveform panel by a vertical line. Thick gray lines represent major sulci as labeled on the lateral hemispheric surface in A. (C) AEPs obtained during PB. Two clusters of recording sites with maximal evoked activity are observed at locations along the superior temporal gyrus anterior and posterior to the transverse temporal sulcus. (LF-lateral fissure, STS-superior temporal sulcus, ITS-inferior temporal sulcus, TTS- transverse temporal sulcus).

**Figure 3:**
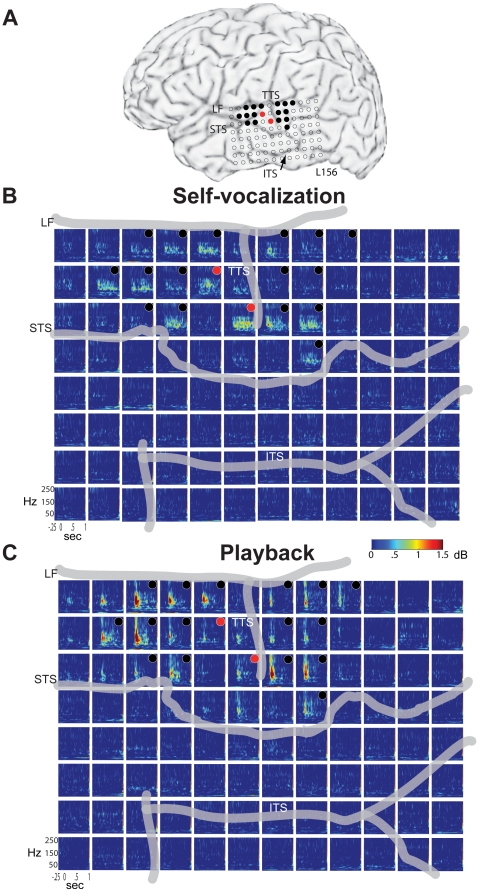
Time-frequency analysis of subject 156's responses during self-vocalization and playback. (A) MRI surface rendering of the left cerebral hemisphere showing the locations of all recording contacts and major sulci. In this figure, filled black circles denote contacts where significant *decreases* (p<.01, 0–500 msec post-stimulus) in high gamma band (HGB, 70–150 Hz) power occurred during self-vocalization (SV) compared to playback (PB). Red circles indicate contacts where significant *increases* in high gamma band power were observed during SV compared to PB. (B) Broad-band time-frequency analysis (2–250 Hz) of evoked responses recorded during SV. Individual panels display the power responses for each frequency band at each recording site (−.25 sec to 1 sec post-voice onset). The largest responses are seen to occur between 70–150 Hz. Thick gray lines represent the major sulci as labeled in A. (C) Time-frequency analysis of evoked responses recorded during the PB condition (LF-lateral fissure, STS-superior temporal sulcus, ITS-inferior temporal sulcus, TTS- transverse temporal sulcus).

Digitized voice signals were recorded simultaneously with the evoked brain responses using the TDT system to provide a common time scale for both the evoked cortical responses and voice signals. Stimulus-evoked potentials were created using a back-averaging method whereby the voice onset of each utterance was manually identified in the sound waveform using a thresholding technique. As the time intervals between the individual utterances were identical for both SV and PB conditions, the same voice onsets identified in the SV task were used for the PB task. From these onsets, individual trials of data blocks were created to evaluate brain activity before and after each voice onset. All individual voice and brain recording trials were manually inspected and discarded if artifacts were noted. The remaining trials were then averaged to create AEPs (Fig. 2B, C) for each electrode in both conditions. For statistical comparison (see below), the brain activity was binned into three analysis windows (AW) including one window prior to voice onset, and two windows after voice onset. The cortical activity recorded in the SV condition was compared to the analogous window in the PB session (see below). The spectral content of the recorded brain activity was analyzed on an individual trial basis using a wavelet transform based on complex Morlet wavelets. Event-related band power (ERBP) was calculated from power measured in the response window relative to baseline power measured in the reference period (−400 to −200 ms) prior to each stimulus onset. This reference period was chosen because it was free of any acoustic signal (i.e. acoustically “silent”), and it preceded voice onset sufficiently that any pre-vocalization brain activity would be expected to occur after this [20]. Furthermore, since the brain activity captured during each individual trial is referenced to a time period immediately preceding that same trial, any changes in the subject's cognitive state (e.g. changes in attention) over the course of 50 trials, is controlled for. Each frequency band power was normalized to the reference period activity within that same frequency band. The results of these single-trial calculations were then averaged and represented as a plot of power on the time versus frequency axis. For further details of this analysis technique, see Oya et al. [33]. The initial analysis included all frequency bands up to 250 Hz; however it was observed that the most prominent power response was in the 70–150 Hz range. For this report, we refer to this frequency range as the high gamma band (HGB), and subsequent ERBP statistical analysis was limited to this frequency band (see Fig. 3).

### Statistical analysis

Statistically significant differences in evoked responses were determined using an analysis of variance method comparing responses recorded during the SV and PB conditions, for each electrode site, and for both AEP and HGB power responses. In the current experiments, the dependent AEP (or HGB power) measurement was treated as a multivariate response and assumed to be sampled from a multivariate distribution [34]. In this way, MANOVA is a suitable statistical test to determine whether the measured response (i.e. AEP or HGB power) is different between the two conditions. A detailed description of how this approach is used to statistically analyze field potential responses recorded from intracranial recording contacts is provided in a previous publication [32]. Briefly, a three-way repeated measures MANOVA was used to determine if there were differences (Stimulus: SV and PB, analysis window, recording contact) in the AEPs and ERBP recorded during the two conditions. In MANOVA, when the classification has more than one factor, and omnibus tests for main effects and their combinations are significant, it is common to test (i.e. contrast) the means of each level of each factor and their combinations, adjusting the resulting *P*-values to reflect these multiple comparisons. The MANOVA procedure was preceded by a principal component analysis (PCA) in order to reduce the dimensionality of the data vectors [32], [35]. It is not possible to carry out the multivariate analysis using the original vectors secondary to the high dimensionality. The number of principle components utilized is able to account for the variance while allowing a large reduction in dimensionality of the input vectors. We utilized false discovery rate to correct for multiple comparisons to determine significant differences in both the AEPs and HGB ERBP recorded during the SV versus PB. The locations of the electrode contacts that demonstrated statistically significant differences in AEP and/or ERBP are labeled on the surface rendered brain images.

**Figure 4:**
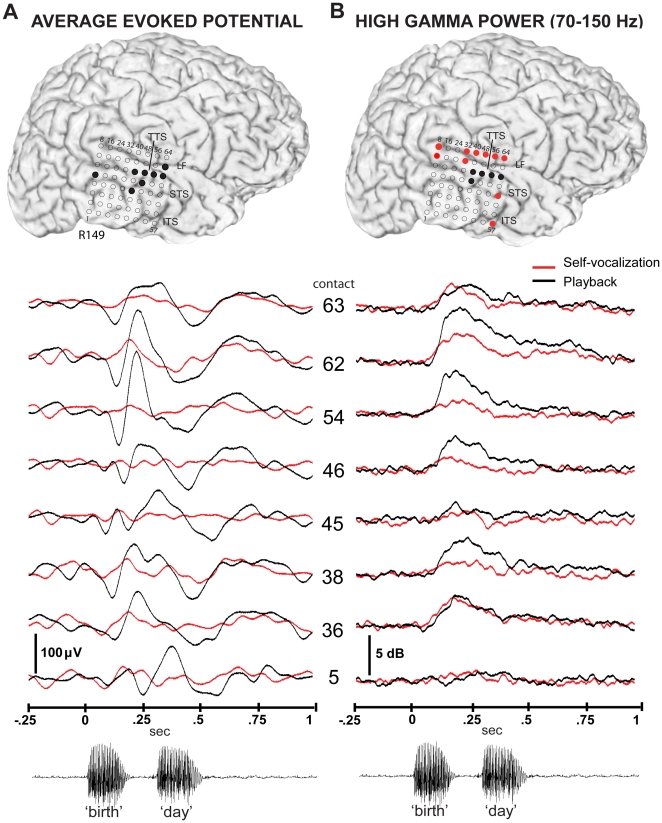
AEP and high gamma power responses in subject 149 during self-vocalization and playback. (A) Right hemisphere surface rendered MRI showing recording site locations and major sulci. Black circles denote contacts where the AEP during the SV condition was significantly attenuated compared to the PB condition (p<.01, 0–500 msec post-voice onset). Tracings below show superimposed AEPs during SV (red line) and PB (black line) recorded from the eight contacts marked with black circles. Contacts are labeled numerically. Onset of vocalizations for both the SV and PB conditions is delineated as time 0. Although responses from all of the displayed channels are attenuated during SV, the magnitude of attenuation and waveform morphologies vary markedly for the different brain sites. A sample acoustic waveform from a representative utterance of “birthday” is displayed below. The same horizontal time scale applies to all panels in this figure. The temporal relationship between AEP waveform morphologies and the acoustic features of vocalization stimuli varies across these brain sites. (B) The same MRI surface rendering as in column A, but with colored circles denoting contact locations where statistically significant changes in high gamma (HGB, 70–150 Hz) power were observed. Black circles indicate contacts with significant *attenuation* of HGB activity and red circles show contacts with an *increase* in the HGB response during SV compared to PB (p<.01, 0–500 msec). Individual tracings below show the averaged HGB power responses for each of the same sites in column A for both SV (red) and PB (black) conditions. The HGB power reduction during SV is seen to vary greatly across the STG, with near complete reduction at some sites (contact 54) and no reduction at others (contacts 36, 63). The duration of the HGB response is seen to vary between these contacts also. (LF-lateral fissure, STS-superior temporal sulcus, ITS-inferior temporal sulcus, TTS- transverse temporal sulcus).

Two different analysis window durations were utilized when making statistical comparisons between brain responses during the SV and PB conditions. The AEPs observed on STG were found to have a polyphasic morphology with components extending to 500 ms beyond the onset of the utterances (e.g. Figs. 1 and 4A), and the average duration of the utterances was 500 ms (middle row, fig. 1). For these reasons, a 500 ms time window was used for some analyses. The HGB ERBP responses were consistently of shorter duration than the AEPs, therefore we also made use of a 250 ms time window to statistically analyze the brain responses. The analysis windows used are indicated in each figure legend.

## Results

Analysis of the electrophysiological data using both AEPs and HGB ERBP has revealed consistent response patterns throughout our series of ten subjects. Findings from a representative subject with electrodes over the left, language-dominant hemisphere are presented in figure 2 (L156, Fig. 2A). During self-vocalization (SV), marked attenuation of AEPs was seen on the STG, as compared to the AEPs obtained during playback (PB) of the same vocalizations (Fig. 2B, C). As indicated by the filled circles, responses recorded from multiple STG sites demonstrated significant differences between the AEPs recorded during the two conditions in the 500 msec period after voice onset (p<.01, PCA-manova). These sites localized in two distinct spatial clusters: one anterior and one posterior to the lateral termination of the transverse temporal (Heschl's) sulcus (TTS). This sulcus is mostly on the supratemporal plane and marks the boundary between the posterior-most transverse temporal gyrus and the planum temporale [36], [37]. In this particular subject, some STG sites positioned between these two clusters showed no significant effects of vocalization, and no sites outside of the STG demonstrated significant AEP differences between the SV and PB conditions.

Examination of this subject's ERBP responses demonstrated a similar pattern of anatomic localization of sites showing significant changes across the two conditions (Fig. 3A). Again, two STG areas where significant response changes occurred were separated by sites without significant changes. Most of the STG sites showing significantly different ERBP responses demonstrated largely an “onset” response to the PB stimuli (Fig. 3C) with strong increases in total power primarily in the HGB, compared to the pre-stimulus baseline power. The HGB onset response was found to be absent, or markedly attenuated during the SV block (Fig. 3B). One possible explanation for why ERBP onset responses were larger than offset responses is that the trials were aligned at voice onset for the purposes of averaging, and the durations of the individual utterances vary from trial to trial. However, this duration variability factor was mitigated somewhat in our experimental paradigm since every utterance was recorded and subsequently played back during the PB block. Therefore, even though there was variability in the offsets of the acoustic stimuli, this variability was identical between SV and PB blocks. In addition, some STG sites demonstrated a sustained increase in high gamma power throughout the vocalization during the SV block that was not seen during the PB block (red circles, Fig. 3A; see also below). The sustained high gamma response pattern seen during SV, but not during PB, likely reflects cortical physiological events that occur during vocalization and are restricted to certain small areas within auditory cortex. Taken together, this subject's ERBP findings demonstrate that within the lateral STG, acoustically responsive cortex might be functionally parcellated into circumscribed cortical regions with distinct physiological responses during vocalization.

All subjects with grids that covered adequate portions of the STG anterior and posterior to the lateral boundary of the TTS demonstrated a discrete area of activation where either the AEP or ERBP responses varied significantly between the SV and PB conditions. Findings from a subject with right hemisphere, non-language dominant electrode implants, and partial STG coverage, are shown in figure 4 (R149; Fig. 4A). Similar to subject L156 (Fig. 2), there was a cluster of sites that showed significantly different responses during the SV and PB conditions located just posterior to the lateral margin of the TTS. This patient's recording array did not cover cortex anterior to the TTS where we would hypothesize an additional cluster is presumably located. AEP waveforms recorded from each of the locations that showed responses that were significantly different for the two conditions are shown in Figure 4 (Fig. 4A). The magnitude of vocalization-induced attenuation varied for these different sites (Fig. 4A). For example, the AEP recorded from contact 54 was essentially completely absent during the SV condition, compared to the PB condition. Yet, other nearby sites showed partial preservation of some AEP peaks (contacts 36, 38, 45, 62) during vocalization. Note that the AEP waveform morphology differed also between the sites during the PB condition. This variability observed in both AEP morphology and degree of attenuation of individual AEP components obtained from auditory cortical recording sites separated by only a few millimeters precludes a meaningful systematic measure of “percent attenuation” or “grand-averaging” techniques utilized in other non-invasive vocalization- playback studies to make generalizations across subjects.

Variations across brain sites were also seen in the high gamma responses in this subject (R149, Fig. 4B). The same locations that demonstrated significant attenuation of AEPs (Fig. 4A) showed a varying degree of HGB power attenuation (Fig. 4B). Responses obtained by contact 54 showed the largest degree of HGB power attenuation, while nearby locations (contacts 36, 45, 63) showed little change in HGB power between the SV and PB conditions. In addition, some locations showed an increase in HGB power during SV compared to the response during the PB condition, and many of the sites showing this pattern of responses were located outside the STG (red circles, Fig. 4B).

**Figure 5:**
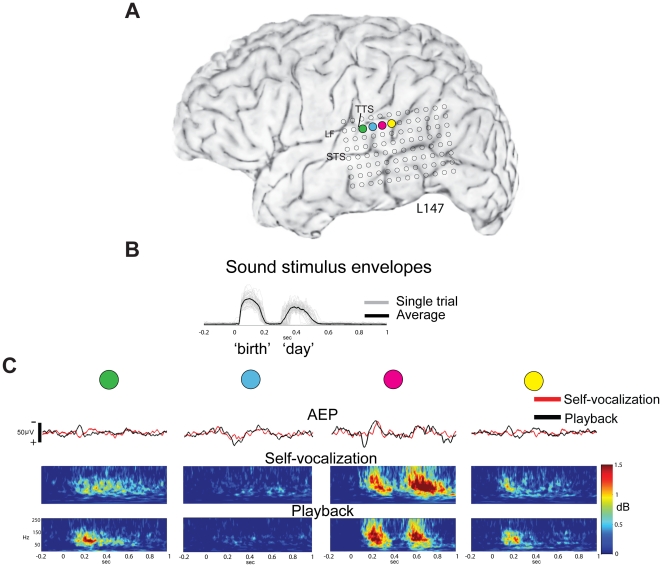
STG evoked responses demonstrate very localized effects of vocalization on speech-sound processing. (A) Surface rendered MRI of the left hemisphere of subject 147. Recording contact locations are depicted by open circles. The four colored contacts are positioned over auditory area PLST and were selected to demonstrate the spatial distribution of vocalization-induced effects on sound processing. The center-to-center distance between the contacts is 5 mm. (B) The individual trial sound stimulus envelopes (gray lines) and the average of all utterances (black line) of the utterance ‘birthday’ are displayed, with time 0 denoting onset of the first syllable. (C) Neural responses recorded from the four recording sites as labeled in A, with AEPs in the top row, and time-frequency spectrograms during SV (middle row) and PB (lower row). The most anterior of the four contacts (*green* circle) shows attenuation in the average evoked response during SV (red line) compared to PB (black line) and HGB attenuation of the onset response but a sustained increase in HGB activity during SV compared to PB. Five millimeters posteriorly, the AEP recorded from the *blue* contact is minimally affected by vocalization, and there are minimal HGB responses during either SV or PB. The largest amplitude AEP is observed at the *magenta* contact, and the initial positive deflection in this response is completely attenuated, while the later negative deflection is slightly delayed but the amplitude is preserved during SV compared to PB. Large increases in HGB power were observed in response to each of the two syllables in the stimulus during both the SV and PB conditions. The most posterior of the four contacts (*yellow*) shows AEP attenuation during SV, and minimal attenuation of the HGB response, which is only an onset response during both SV and PB, and markedly different than the responses from the neighboring contact 5 mm anterior (magenta). (LF-lateral fissure, STS-superior temporal sulcus, TTS- transverse temporal sulcus).

Regional STG response differences, with clear variation seen between sites located only millimeters away from each other, were observed in all subjects. Figure 5 shows another example of regional response differences within STG in a left, language-dominant hemisphere subject (L147, Fig. 5A). The stimulus sound waveforms are shown to illustrate the temporal characteristics of the two separate syllables of the utterance “birthday” (Fig. 5B). The resulting evoked-responses obtained from four closely-positioned STG contacts collectively covering a cortical expanse of only two centimeters demonstrated markedly different response patterns to the same acoustic stimulus. The activity recorded from the first site (green circle, Fig. 5B) showed marked attenuation of the AEP during SV, as well as attenuation of HGB ERBP. This site was located just anterior to the TTS, and showed a sustained HGB response throughout the utterance during SV, but only an onset HGB response during PB. A neighboring, more posteriorly-located site (red circle, Fig. 5B), demonstrated a markedly different response type. The AEP from this posterior location showed absence of early peaks, with partial preservation of later peaks. Differences observed in the ERBP response to the stimulus were even more striking—this site demonstrated a clear capacity to follow each syllable in the two-syllable utterance during both SV and PB, with only a slight attenuation of HGB activity seen in the response to the first syllable (Fig. 5B). No attenuation occurred in the response to the second syllable. A fourth site, located only 5 mm posterior to this location illustrated yet another response type (yellow circle, Fig. 5B). Both AEP and HGB ERBP attenuation was seen, but this posterior-most location only demonstrated an onset HGB response. While this site shows onset responses to both SV and PB, there is a subtle difference in latency seen in the HGB responses, with the earlier HGB response to SV preserved but a later response slightly attenuated compared to the PB condition.

**Figure 6:**
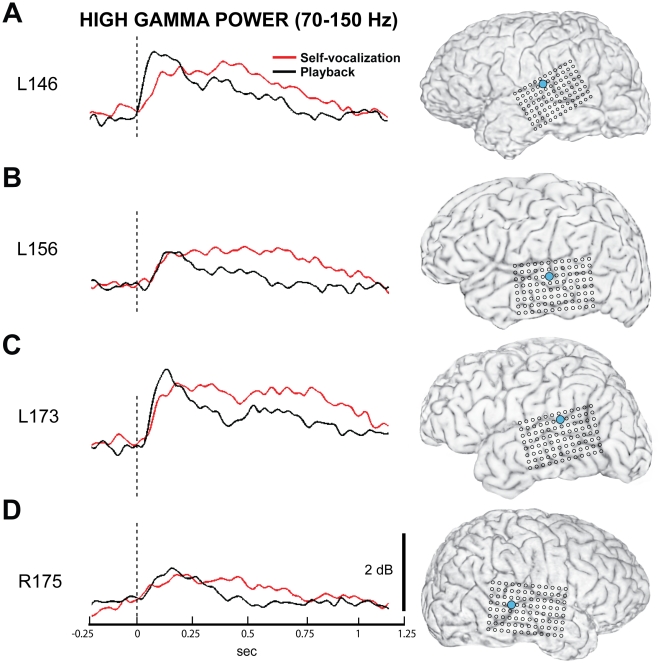
Some sites within auditory cortex demonstrate increased HGB activity during self-vocalization compared to playback. These are four example subjects as labeled (A–D, left column) with the surface rendering of each subject's MRI with the recording site indicated (filled blue circle, right column). In each subject, these brain sites demonstrated increased averaged HGB power responses during SV (red waveforms, middle column) compared to responses obtained during PB (black waveforms, middle column). These HGB responses were ‘sustained’ throughout and beyond the duration of the utterance during SV, while the PB HGB responses were more consistent with an ‘on’ response.

Every subject in this series had at least one site on lateral STG that demonstrated either an increase in HGB power during SV compared to PB, or a categorical change in HGB response type with an “onset” response during PB and a “sustained” response during SV. Exemplars of such response-type changes are shown in figure 6. In these 4 examples, the stimulus-evoked increase in HGB power occurred during a longer time period during SV, compared to the PB condition.

**Figure 7:**
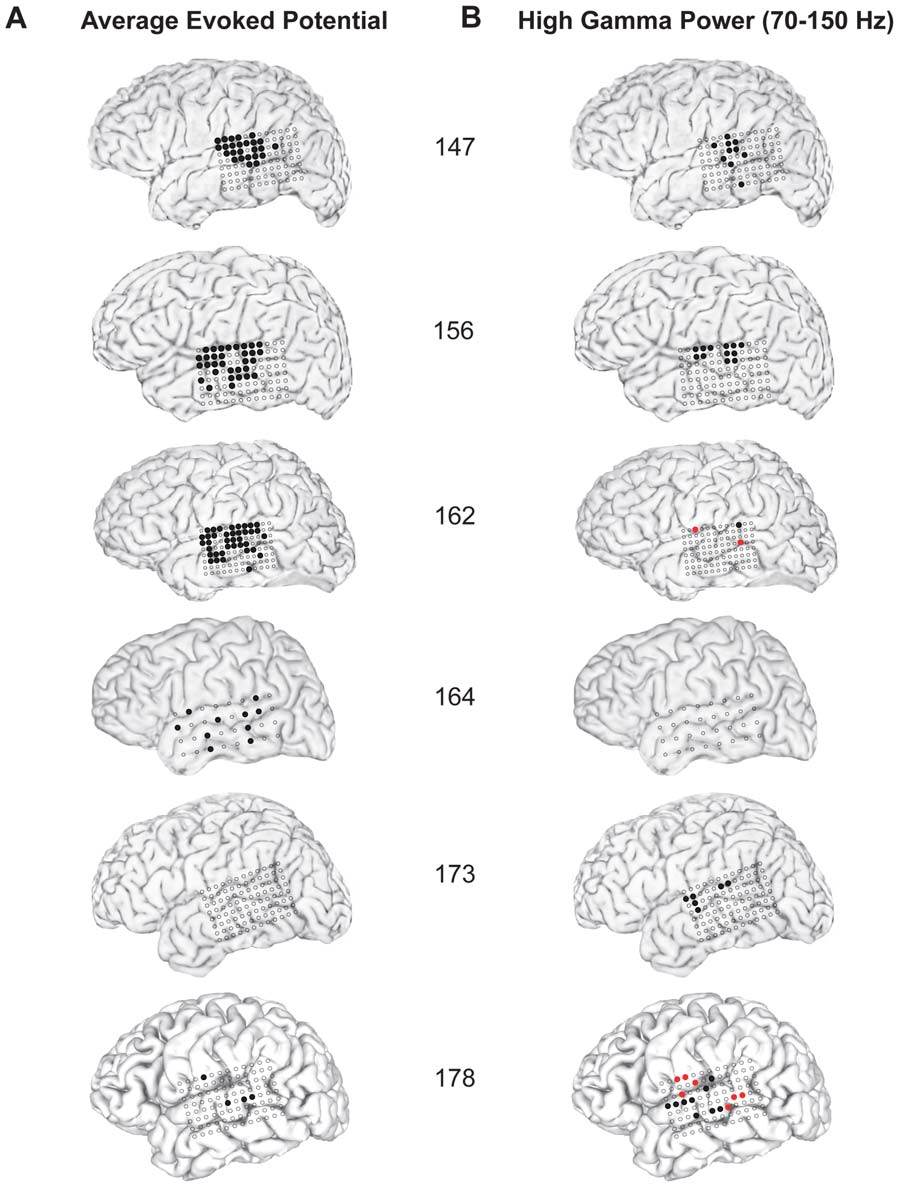
Summary analysis of left-sided subjects. MRI surface renderings of subjects with left-sided implants (n = 6) demonstrating the locations of recording sites (filled circles) where evoked responses differed significantly during the SV versus PB conditions during the first 250 ms following stimulus onset. Contacts that demonstrated significantly attenuated AEPs during the SV condition (filled black circles, A, left column) were most often located over the lateral surface of the superior temporal gyrus. Recording sites that showed significant attenuation in the HGB power response during SV (filled black circles, B, right column) also were most often located over the superior temporal gyrus. Proportionally fewer sites demonstrated HGB responses that were significantly larger for the SV condition compared to the PB condition (filled red circles, B), and the locations of these sites did not conform to a consistent topographic pattern relative to gross anatomical landmarks of the lateral hemispheric surface.

**Figure 8:**
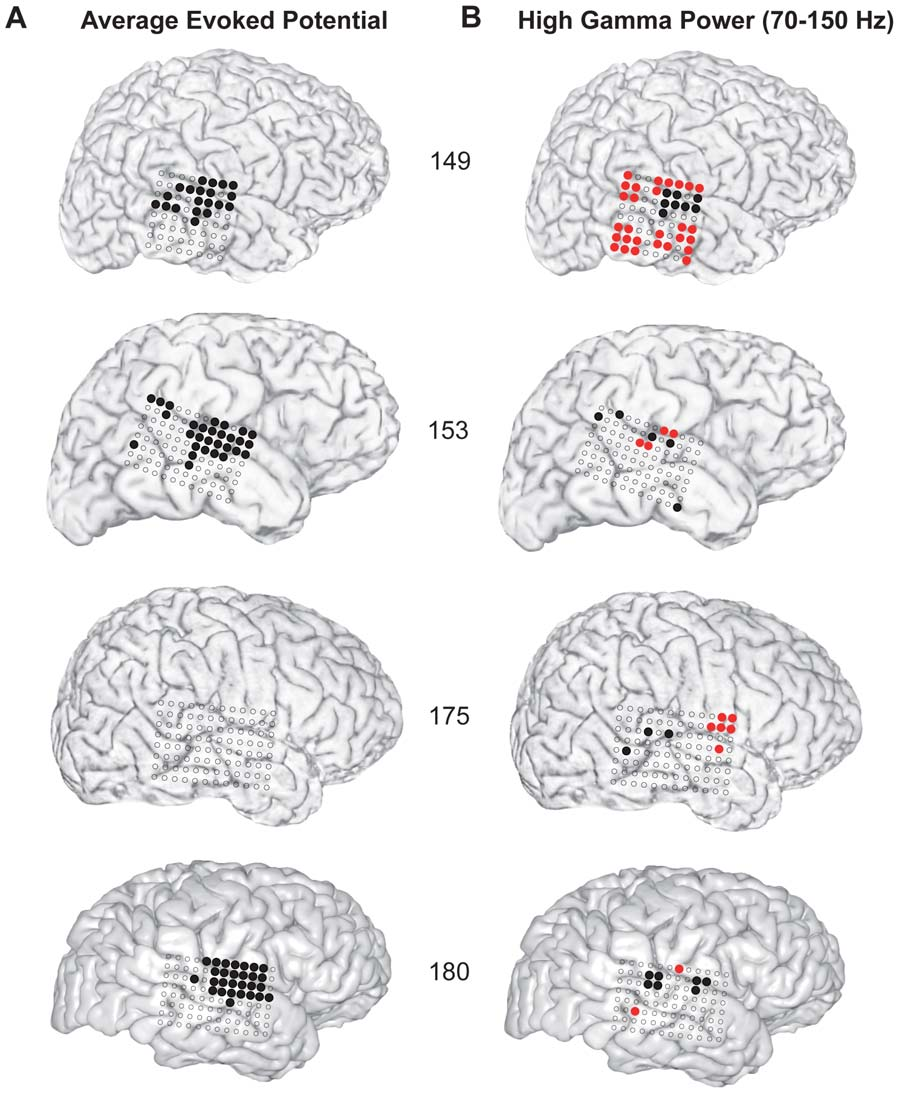
Composite analysis of right-sided subjects. Lateral hemispheric surface renderings of right-sided subjects (n = 4) showing recording sites where responses were significantly different for the SV versus PB conditions (filled circles) during the first 250 ms following stimulus onset. In three of the four subjects shown, a large number of STG contacts showed a statistically significant *decrease* in AEP responses during SV (panel A, filled black circles), and there were no sites where responses were increased during SV. Sites showing statistically significant differences in HGB power responses for the two conditions are shown in panel B (filled circles). In all four subjects STG sites were identified where HGB power *decreased* significantly during SV (filled black circles, panel B). With the exception of subject 175, a smaller number of recording sites show a significant reduction in HGB power compared to the number of sites showing reductions in AEP responses. In all right hemisphere subjects, sites were identified where significant *increases* in HGB power occurred during SV (red circles). These sites were observed in different STG and non-STG locations that did not conform to a consistent anatomical pattern across subjects.

Results evaluated across the entire subject series (N = 10) demonstrate an overall similar pattern of responses along the lateral STG. In both left (Fig. 7) and right (Fig. 8) hemisphere subjects, both in the AEP and ERBP evoked responses, there were areas on the posterolateral STG where responses differed significantly between the SV and PB conditions. As seen in these figures, the contacts showing such response differences were most often located on the portion of the STG near the lateral termination of the TTS. In the left sided AEP analysis, there is a suggestion of two response areas separated by the TTS (Fig. 7A), while this separation is not apparent in the right-sided subjects (Fig.8A).

The response patterns seen across these 10 subjects show that there are a larger number of temporal lobe sites demonstrating significant vocalization-induced changes in the AEP responses than sites that show significant HGB power changes (Figs. 7A,8A). The sites where HGB power responses are reduced during SV most often localize to the middle and posterior STG. There are also contacts on STG, and outside of STG that show increased high-gamma power during SV compared to PB (red circles, Figs. 7B, 8B). The locations of these sites did not conform to a consistent anatomical pattern across subjects.

There is marked variability in the proportion of recording sites capturing responses that were significantly different for the SV and PB conditions. The degree to which specific recording results vary across experimental subjects likely results from small differences in electrode grid locations and the known inter-subject variability in the locations of specific auditory fields relative to gross anatomical landmarks. In some subjects, and during some experiments, the signal-to-noise ratio of auditory evoked responses to all classes of auditory stimuli was reduced as a result of increased electronic noise levels. In other instances, the number of effective stimulus presentations during an experimental session was reduced as a result of post-hoc rejection of artifact contaminated epochs. These factors contributed to a relative loss of statistical power in experiments performed in left hemisphere subjects 164, 173, and 178 and right hemisphere subject 175. In addition, subject 173 demonstrated overall diminished auditory cortical responses to other acoustic stimuli (e.g. clicks, tones) during other experimental sessions.

## Discussion

The results of the current experiments provide the first directly recorded electrophysiological evidence of vocalization-induced activity associated with speech-sound processing within human auditory cortex. The effects were predominantly suppressive in nature, consistent with reports from earlier experimental animal and non-invasive human studies. New findings include the observations that vocalization-associated effects occur within relatively-circumscribed regions of the lateral superior temporal gyrus, and activation at some STG sites is enhanced during vocalization. Changes were observed in both AEP and HGB power, but the results are not identical for these two different measures of brain activity.

Normal hearing humans continuously make use of auditory information to adjust their vocalizations and optimize speech communication. A wide range of experimental approaches has been used to investigate the neural systems that subserve this sensory-motor integration in humans. One model postulates that when humans vocalize, the vocal motor system produces a motor speech template, or efference copy, that is utilized within auditory cortex to compare the auditory stimuli that is actually heard during vocalization, with the vocalization that the motor system intended to produce [21]. When the acoustic stimulus matches the intended speech signal, the model predicts that the resulting evoked brain activity will be ‘cancelled’, or suppressed.

Our most detailed understanding of how auditory cortical neurons change their firing patterns during vocalization comes from experimental animal studies. In this setting, action potentials generated by individual auditory cortex neurons can be recorded using microelectrodes. This invasive method has been used extensively to study the basic functional properties of auditory cortical neurons in various species; however, it is very difficult to obtain these recordings in awake, vocalizing animals. The first reported microelectrode experiments of this type did not rely on spontaneous vocalizations, but relied instead on electrical stimulation of the brainstem central gray matter to evoke vocalizations in the squirrel monkey [19]. These investigators recorded superior temporal gyrus (STG) neurons during the induced vocalizations, and also when the vocalizations were played back to the animal. A majority of STG neurons displayed decreased firing rates during stimulation-induced vocalization compared to the rates observed during playback. A subpopulation of STG neurons was also identified that displayed response properties that were not altered by vocalization.

Auditory cortical microelectrode recordings in non-human primates obtained during spontaneous vocalizations have only recently been reported [20]. These experiments were carried out in marmosets, a primate species that makes extensive use of vocal communication. When the monkeys spontaneously vocalized, a majority (∼75%) of auditory cortical neurons suppressed their firing rates, and in some neurons this effect began prior to the onset of vocalization. These investigators also described a less frequently encountered type of auditory cortical neuron that exhibited an increase in firing when the animal vocalized. These findings provided the first direct evidence of how auditory cortical neurons alter their firing patterns during spontaneous vocalization and demonstrated that the predominant effects were suppressive in nature.

Investigators face unique challenges when seeking to pursue a similar experimental strategy in humans. Unlike monkeys, humans can be easily trained to perform vocalization tasks. However, safety considerations limit the types of human brain recording methods. Vocalization-induced changes in auditory processing have been characterized using non-invasive scalp EEG and MEG methods [3], [4], [5], [6], [7], [8], [9], [10]. The most consistently reported finding from these studies is attenuation of the averaged evoked response during vocalization, compared to vocal playback. This predominantly suppressive effect of vocalization on the auditory evoked response is generally consistent with findings in experimental animals and a forward model of vocal control [21]. However, there are inherent limitations in the ability of EEG and MEG to accurately localize brain activity, which preclude resolving vocalization-induced effects with the same resolution as microelectrode studies.

A number of lines of evidence suggest that the effects of vocalization will differ for different areas of human auditory cortex and that high-resolution recording methods are required to characterize this organizational pattern. Extrapolating from anatomical and physiological data derived from experimental animal studies, and more limited human studies, it is hypothesized that human auditory cortex is comprised of ten or more fields organized into core, belt and parabelt groups [38], [39], [40]. These fields are postulated to have distinct functional properties and patterns of anatomical connectivity. If the functional connections known to exist in non-human primates between frontal lobe motor control areas and temporal lobe auditory cortex also exist in humans, activation of these pathways would be expected to differentially influence auditory processing in fields outside of the core area [41], [42]. These patterns of fronto-temporal connectivity are considered in a theoretical model of vocal motor-sensory integration proposed by Guenther and colleagues [22], [24], [43]. In this model, speech-sound information generated within the frontal lobe is projected to higher-order auditory areas within the STG. Findings from earlier functional imaging studies are consistent with this hypothesis that vocalization effects are most pronounced in circumscribed regions of higher-order auditory cortex [13], [44].

The current experiments were designed to examine this hypothesis directly using the opportunity to record from auditory cortex on the lateral STG of neurosurgical patients. By recording brain activity using electrode arrays positioned on the pial surface it is possible to examine responses with a high degree of spatial and temporal resolution. The results consistently demonstrated circumscribed areas of cortex along the lateral STG where responses differed during the self-vocalization and vocal playback conditions. The most consistently identified area was overlying the lateral terminus of the transverse temporal (Heschl's) sulcus (TTS). In this area, the amplitude of AEPs and HGB power were most often diminished during the SV condition. In some cases, there was intervening cortical tissue within this area of the STG that was acoustically responsive, but not significantly altered by vocalization.

Although the dominant vocalization-related effect was *suppressive* in nature, there were also clear examples of small areas of STG where HGB power was markedly *increased* during SV compared to PB. In those instances, the vocalization-induced increase in HGB power was not accompanied by amplitude increases of the average evoked potential and the effects of vocalization were only detected by analyzing bandpass power changes. This finding emphasizes the importance of analyzing both the phase-locked and non-phase locked activity recorded from intracranial electrodes, as previously reported by Crone and others [45], [46]. The underlying cellular events that generate the observed changes in HGB power cannot be determined with certainty, however recent findings from experimental animal studies indicate that high frequency power changes more closely reflect auditory cortex tonotopic patterns determined using microelectrode recordings than do average evoked potentials [47]. In addition, auditory short-term memory processing has been reported to affect gamma band activity [48].

One of the questions that investigators seek to address with vocalization-feedback experiments is *when* vocalization-associated effects occur within auditory cortex. Single unit recordings obtained in marmoset auditory cortex clearly demonstrate that suppressive effects of vocalization begin more than one hundred milliseconds prior to onset of vocalization [20]. The results of scalp EEG and MEG experiments performed in humans demonstrate shifts in the latencies of some event related potential waveform components during vocalization, compared to playback [9], [49]. Because of significant methodological differences between the current experiments and these previous studies it is difficult to compare results from these different experiments as they pertain to the timing of vocalization effects [3], [4], [5], [8], [9], [10], [50].

The recording montages used during MEG and EEG experiments are standardized across subjects and measure the summed activity of large populations of neurons. With these methods the waveform morphologies of auditory evoked potentials are well characterized with features that can be reliably identified and compared across subjects, and investigators have described vocalization-associated changes in amplitude and latency of the averaged auditory evoked potentials. This same approach is less well suited to the analysis of the current data set for a variety of reasons. The first is the large magnitude of the vocalization effect observed in the current study. In contrast to non-invasive studies where modest changes are observed in the averaged evoked waveforms, many of the responses recorded directly from STG are entirely absent during vocalization, or so severely attenuated that waveform components cannot be compared across the SV and PB conditions. The second factor that complicates this analysis is the high degree of variability observed in the AEP waveforms recorded from different sites along the STG. The AEP waveform recorded from the focus of maximum response within area PLST has been described previously and can be consistently identified across subjects, but this represents only a small portion of the STG from which auditory evoked responses are obtained in the current experiments [31]. Anterior and posterior to PLST the AEP waveforms are highly variable, thus limiting our ability to make brain-site to brain-site comparisons across subjects.

Vocalization was also associated with alterations in the temporal patterns of HGB changes. These findings cannot be directly compared with previous studies because this is the first report where HGB power was directly measured in a vocalization-playback experiment using the subject's own voice. The temporal patterns of HGB power changes were complex and varied by vocalization condition, and location along the STG. The duration of the power changes also varied significantly for different brain sites and conditions. One commonly observed response type was characterized by a transient increase in HGB power soon after stimulus onset, consistent with an ‘*on*’ response. A different, ‘*sustained*’ HGB response pattern was also observed whereby increases in power were maintained throughout the duration of the vocalization. At brain sites where vocalization was associated with diminished HGB power, this suppression was typically manifest as a decrease in the magnitude of the power throughout the response, without an obvious change in the overall temporal pattern of the response (Fig.4). In contrast, at the sites where HGB power increased, the temporal pattern of the responses was altered as well. At these sites, an ‘on’ response was observed in the PB condition, and this changed to a ‘sustained’ response pattern during self-vocalization (Fig.6). The striking differences between these two response types suggests that fundamentally different mechanisms of cortical processing are activated during the SV and PB conditions at these brain sites, as opposed to a graded modulation of the same activation process.

In almost all cases, the onset of HGB power changes occurred after stimulus onset. In rare instances, power changes occurred prior to vocalization, but these sites did not conform to a consistent anatomical pattern across subjects and the significance of this finding is uncertain. This observation contrasts with the unambiguous findings in marmosets where suppression of neuronal firing was observed prior to vocalization onset [51]. It is possible that pre-vocalization changes were not consistently observed in the current study because of the recording method used. Action potential firing cannot be directly measured using the recording techniques employed in the current study. Another variable to consider is the region of auditory cortex studied. Our recordings were obtained from higher-order auditory cortices, whereas the marmoset recordings included core cortex.

A number of caveats must be considered when interpreting the results of the current experiments. Invasive recordings in humans allow investigators to record electrophysiological events with a degree of combined spatial-temporal resolution that cannot be achieved using non-invasive experimental approaches. However, the intracranial electrodes cover only a portion of auditory cortex. The effects of vocalization on auditory processing within presumed core and belt fields located in the supratemporal plane cannot be studied using electrodes positioned over the lateral STG. Also, direct recordings are only obtained from one hemisphere in each subject, precluding the ability to make within-subject comparisons of simultaneously recorded responses in right and left hemispheres.

There are also limitations inherent to the SV versus PB experimental protocol irrespective of the brain recording method used. One is the assumption that the subject hears the same acoustic stimulus during both SV and PB conditions. In fact, the acoustic signals activating the cochlea are not identical in the two conditions. During vocalization, a portion of the total acoustic signal is conducted through bone and soft tissue and is attenuated and spectrally filtered before it reaches the cochlea. This bone-conducted signal cannot be precisely measured and therefore cannot be exactly replicated during playback [52]. Sound intensity is known to impact brain responses [28], [29], [30] so in a subset of experiments, the same playback stimuli were presented at different sound intensities to examine how the evoked responses changed when stimuli ranged from being “softer” to clearly “louder” than what the subject heard during vocalization. The response patterns were not significantly altered as a function of the sound intensities used, indicating that bone conduction effects are unlikely to have significantly influenced the overall findings (Fig.1).

Another limitation of the SV versus PB experimental design is that only certain aspects of the forward model are tested. The data in the current report, for example, provide no information concerning the stimulus specificity of the vocalization-associated alterations observed within auditory cortex. Other investigators, using non-invasive recording methods, have probed the specificity of response changes by altering the acoustic properties of the feedback stimulus and examining how brain responses are affected by induced mismatches between the intended vocalization and the speech signal heard by the subject [9], [49], [53]. We have incorporated this same experimental strategy into our ongoing invasive recording studies and will address these findings in future reports.
